# Multiscale modeling of acute insulin resistance in critical care

**DOI:** 10.1186/cc12392

**Published:** 2013-03-19

**Authors:** A Pritchard-Bell, G Clermont, B Yegneswaran, R Parker

**Affiliations:** 1 University of Pittsburgh, PA, USA; 2 University of Pittsburgh Medical Center, Pittsburgh, PA, USA

## Introduction

Stress hyperglycemia in the critically ill is a complex process in which insulin signaling is systematically hijacked to provide energy substrate for metabolic priorities such as cell healing or infection containment. Fluctuating levels of plasma glucose are associated with increased mortality in the ICU [1]. We develop a multiscale mathematical model that can characterize the severity of stress hyperglycemia based on a fundamental understanding of the signaling molecules involved.

## Methods

Insulin resistance following insult has been shown to be driven primarily by the immune response via the cytokine IL-6 [2]. We created a multiscale mathematical model that links circulating glucose and insulin concentration dynamics from the extended minimal model [3] to a cellular insulin response model [4] that captures insulin-mediated glucose uptake in an insulin-responsive cell.

## Results

**Figure 1:**
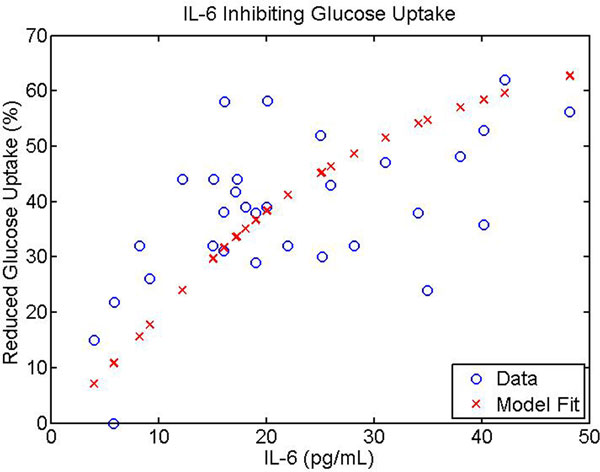
**Reduced glucose uptake driven by increased plasma IL-6 levels**.

Inhibitory dynamics driven by IL-6 were incorporated into the cellular model to attenuate an insulin signaling intermediate (insulin receptor substrate 1) according to the proposed biological mechanisms. The percentage reduction in glucose uptake as a function of IL-6 concentration was fit to data from patients who underwent elective abdominal surgery [2], shown in Figure 1. The overall multiscale model captures decreased insulin signaling as a result of increased IL-6 levels and the subsequent hyperglycemia that may ensue.

## Conclusion

A multiscale model has been developed to describe the inhibitory effects of IL-6 on insulin-mediated glucose uptake. Cellular inhibitory dynamics were shown to capture reduced insulin sensitivity on the macroscale, which could then be used to characterize insulin sensitivity and to provide insulin treatment advice to reduce glucose variability.
